# A Pilot Convenience Survey of Clinicians’ Perception of the Desirability of a Lower Dose Option of an Opioid Analgesic: Hydrocodone as an Example

**DOI:** 10.7759/cureus.106039

**Published:** 2026-03-28

**Authors:** Claudio S Pergolizzi, Joseph V Pergolizzi, Thomas Harrison, Kimberlee A Piazza, Morgan Wagner King, Robert B Raffa

**Affiliations:** 1 Project Management, NEMA Research Inc., Naples, USA; 2 Anesthesiology, NEMA Research Inc., Naples, USA; 3 Data Management, NEMA Research Inc., Naples, USA; 4 Project Management (Intern Program), NEMA Research Inc., Naples, USA; 5 Pharmacy, Temple University (Emeritus), Philadelphia, USA

**Keywords:** convenience survey, dosage strength, dosing flexibility, hydrocodone, opioid prescribing

## Abstract

Healthcare providers (HCPs) need to balance the need for effective analgesia with opioids against the risks associated with opioid therapy. As just one example, current formulations of one of the most commonly prescribed opioids in the United States (hydrocodone) are limited to a minimum strength of 10 mg. However, in clinical settings, providers often encounter patients who may benefit from more individualized and flexible dosing options. A lower-dose opioid formulation might be desirable: to help optimize pain control, reduce adverse events, or ease concerns about opioid-related abuse risks. To better understand provider perspectives, prescribing practices, and the perceived benefits of lower-dose opioid options, a targeted survey of healthcare professionals was conducted. The goal was to capture real-world insights into current opioid pain management options and evaluate the potential role of a lower dosage strength. In order to ground the survey in the participant’s clinical experience, a specific example was chosen. The objective of the current survey was to assess healthcare providers’ perspectives on current opioid analgesic prescribing practices and, as a practical example, to specifically evaluate interest in a potential lower-dose (7.5 mg) hydrocodone formulation. The survey included physicians, nurse practitioners, physician assistants, and other clinicians. The results of the survey indicated a high interest in a lower-dose hydrocodone formulation, with an average rating of 7.9 out of a possible 10, with nearly two-thirds of participants selecting a rating of 9 or 10 out of a possible 10. The respondents identified the key perceived benefits of the availability of a lower dose as follows: more flexible dosing (20 (42%)), fewer adverse events (11 (23%)), and less fear of causing opioid use disorder (OUD) (2 (4%)). About one-third of respondents (17 (35%)) indicated that all three were perceived benefits. These findings suggest that expanding opioid dosing options could support individualized pain management strategies across a range of specialties. Further research in larger and more diverse provider populations is warranted to confirm these insights and guide potential recommendations and implementation.

## Introduction

There are clinical pain settings for which opioid analgesics are the best option [1-4]. However, well-known dose-related opioid-induced adverse effects (e.g., nausea, constipation, drowsiness, dizziness, and respiratory depression) and fear of triggering an opioid use disorder (OUD) are major concerns. Other reasons could relate to demographic differences (e.g., age and sex), genetics (e.g., drug-metabolism enzyme polymorphisms), or individual sensitivity [5-8]. In an effort to provide individualized dosing to address this dilemma, healthcare providers (HCPs) may confront the problem that approved formulations are limited to doses higher than what they desire for a specific patient [9,10]. The widely prescribed opioid analgesic hydrocodone provides such an example of an effective pain reliever that has a typical dose-related incidence of opioid-induced adverse effects [11]. The Centers for Disease Control and Prevention (CDC) and the American Academy of Pain Medicine recommend initiating opioid treatment at the lowest possible effective dose, but 10 mg is currently the lowest single-entity dose on the US market. The registry ClinTrials.gov does not list any applications for trials to study lower-dose formulations of single-entity hydrocodone.

Single-entity hydrocodone (bitartrate) is currently marketed as extended-release (ER) capsules and tablets, intended for the management of severe, chronic pain. It is available in 10, 15, 20, 30, 40, and 50 mg 12-hour ER capsules, as well as 20, 30, 40, 60, 80, 100, and 120 mg 24-hour abuse-deterrent tablets. Hydrocodone is available in lower doses of 2.5, 5, and 7.5 mg, but only in combination with non-opioid analgesics such as acetaminophen (paracetamol) and the nonsteroidal anti-inflammatory drug (NSAID), ibuprofen, which have their own associated adverse event profiles, such as increased risk of liver damage with acetaminophen or gastrointestinal bleeding with an NSAID, that the HCP might wish to avoid [12,13].

To better understand the level of HCP’s desire for lower-dose opioid options, a targeted survey was conducted. In order to capture a focused, not nebulous, opinion, the specific example of hydrocodone was selected. The primary objective of this survey was to assess HCP’s perspectives on current analgesic prescribing practices and to evaluate interest in a potential 7.5 mg ER hydrocodone formulation. Specifically, the survey aimed to identify respondent specialties and prescribing patterns for analgesics; assess the extent of opioid versus non-opioid use, as well as combination prescribing; measure the level of interest in a 7.5 mg hydrocodone formulation compared with currently available strengths; and understand perceived clinical benefits of introducing a 7.5 mg hydrocodone dose, considering flexibility, safety, and patient outcomes.

## Materials and methods

This study utilized a cross-sectional in-person convenience survey (N = 48) intended to evaluate HCP perspectives on current analgesic prescribing practices and their potential professional interest in a lower-dose option (7.5 mg) hydrocodone formulation. Participation was voluntary, and all responses were anonymized. No patient data were collected. The protocol was approved by an Independent Ethics Committee (code #PWK-HCP-2025) on August 30, 2025. The survey instrument consisted of multiple-choice and rating-scale items that covered respondent demographics, current prescribing practices, use of hydrocodone, level of interest in a 7.5 mg hydrocodone formulation, and perceived benefits of such a dose.

The survey questions were as follows: Question 1: What is your specialty?, Question 2: Do you prescribe analgesics?, Question 3: If you do prescribe analgesics, what percentage of your current analgesic prescribing involves non-opioid alone, or a combination (opioid + non-opioid)?, Question 4: If you do prescribe opioids, what percentage of your opioid prescribing involves hydrocodone alone versus a combination of hydrocodone and a non-opioid?, Question 5: Rate your interest on a scale of 1 to 10 in having an option of a 7.5 mg hydrocodone formulation (the current lowest available is 10 mg), and Question 6: Which option most closely represents your perceived benefit that a 7.5 mg hydrocodone dose could provide to patients if available?

The survey data were analyzed using quantitative methods. Specifically, descriptive statistics, including frequencies, percentages, and mean ratings, were used to summarize responses across key domains: respondent demographics, prescribing practices, level of interest in a 7.5 mg hydrocodone formulation, and perceived clinical benefits of introducing a lower-dose option. Data were analyzed in aggregate to protect participant anonymity and to identify trends across respondent groups. All analyses were conducted in accordance with the study protocol and aligned with the intended use of results to inform future clinical and research considerations related to pain management.

## Results

The results are presented below by survey question, with both graphical representations and accompanying narrative summaries to highlight key findings.

Question 1: What is your specialty?

**Figure 1:**
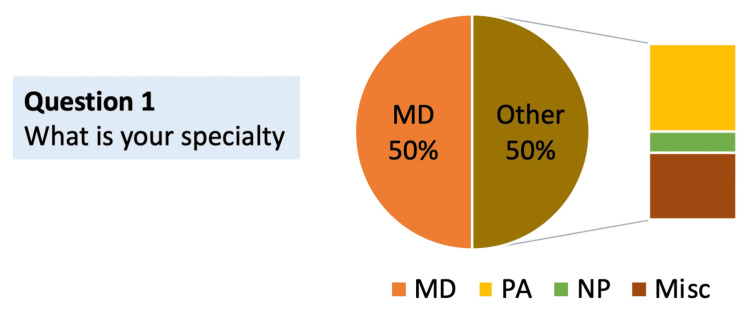
Distribution of respondent specialties (N = 48) MD: doctor of medicine, PA: physician associate/assistant, NP: nurse practitioner, Misc: miscellaneous/others

The responses to this question revealed a broad spectrum of HCP specialties. The majority of the participants were physicians (MD and DO), with strong representation from physician associates/assistants (PAs) and additional contributions from nurse practitioners (NPs), dentists (DMDs), and other HCPs (Figure 1).

Question 2: Do you prescribe analgesics?

**Figure 2:**
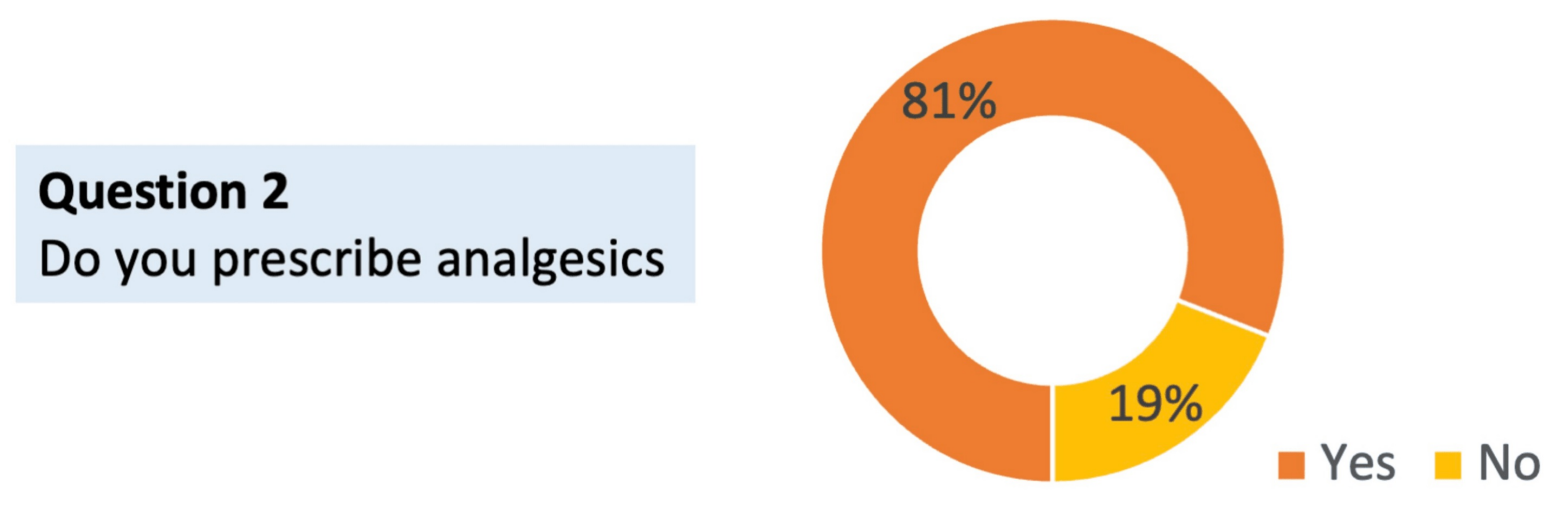
Proportion of respondents who prescribe analgesics Sample size of respondents (with specialties shown in Figure 1) = 48

As expected, the majority of respondents (39(81%)) reported that they currently prescribe analgesics. This demonstrates that most of the participants are actively involved in pain management prescribing practices, ensuring that their insights on opioid and non-opioid use reflect direct clinical experience (Figure 2).

Question 3: If you do prescribe analgesics, what percentage of your current prescribing involves non-opioid alone, opioid alone, or a combination (opioid + non-opioid)?

**Figure 3:**
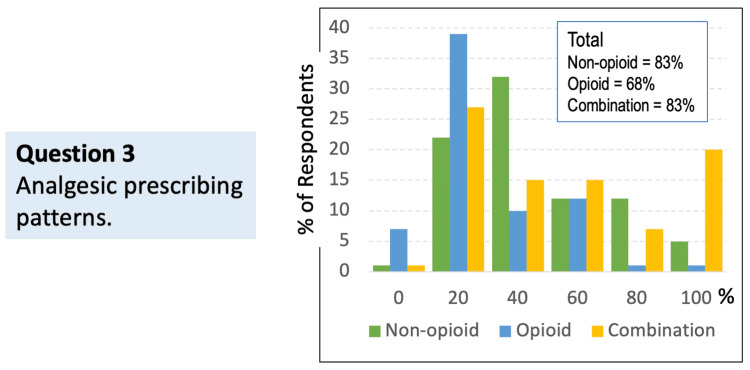
Distribution of analgesic prescribing patterns among providers who prescribe analgesics (N = 48) Respondents could indicate multiple options if more than one applied

Respondents’ prescribing patterns varied across therapy types: non-opioid alone, frequently utilized, reflecting efforts to manage pain without opioids when appropriate; opioid alone, prescribed at a lower rate compared to non-opioid or combination therapy; and combination therapy (opioid + non-opioid), widely reported, indicating that multimodal analgesia remains a common clinical approach (Figure 3).

These findings highlight that although non-opioid prescribing is common, many providers integrate combination strategies in order to optimize pain control while simultaneously mitigating opioid-related risks.

Question 4: If you do prescribe opioids, what percentage of your opioid prescribing involves hydrocodone alone versus a combination of hydrocodone and a non-opioid?

**Figure 4:**
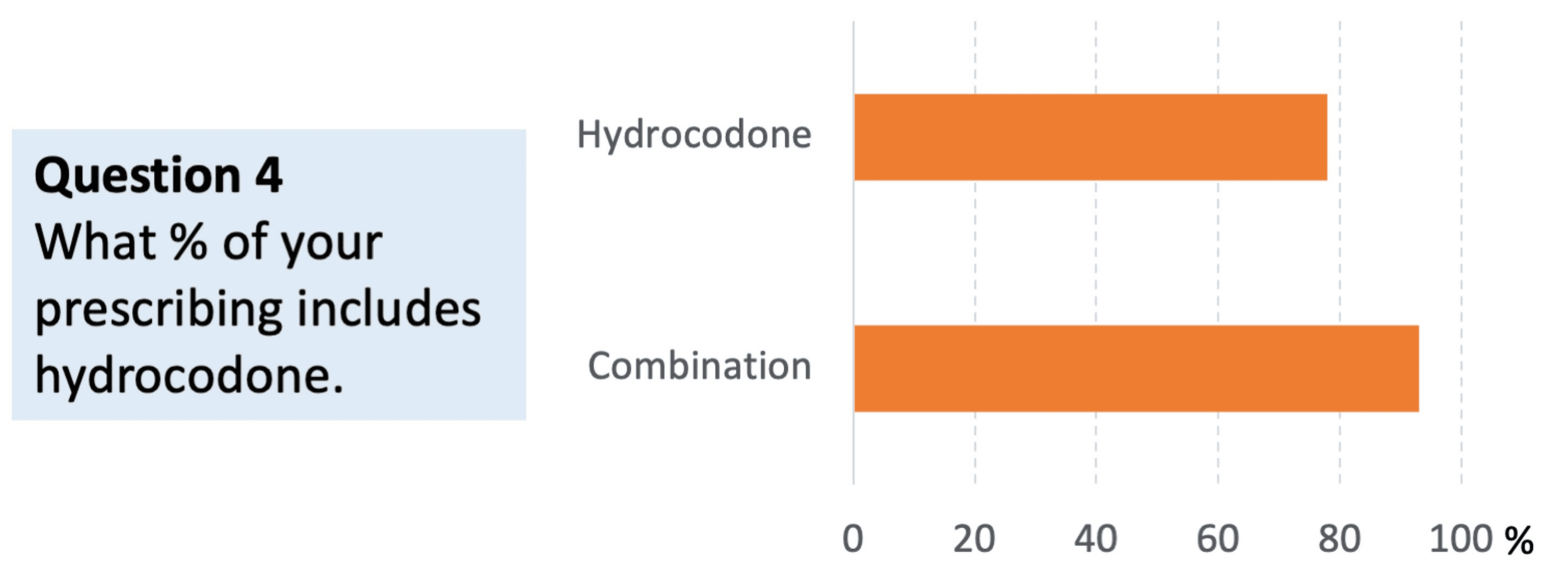
Distribution of hydrocodone prescribing: hydrocodone alone versus hydrocodone in combination with a non-opioid (N = 48) Respondents could indicate instances of hydrocodone used alone and/or in combination with other analgesic(s)

Among respondents who prescribe opioids, the use of hydrocodone occurs both as a standalone therapy and in combination with non-opioids. The results indicate that while hydrocodone alone is prescribed, many respondents favor combination therapy approaches, reflecting an emphasis on multimodal pain management strategies (Figure 4). This pattern is consistent with the common use of hydrocodone in clinical practice but also highlights the use of adjunctive non-opioid therapies to mitigate concerns about opioid-related adverse effects and other problems.

Question 5: Rate your interest on a scale of 1 to 10 in having an option of a 7.5 mg hydrocodone formulation (the current lowest available is 10 mg)

**Figure 5:**
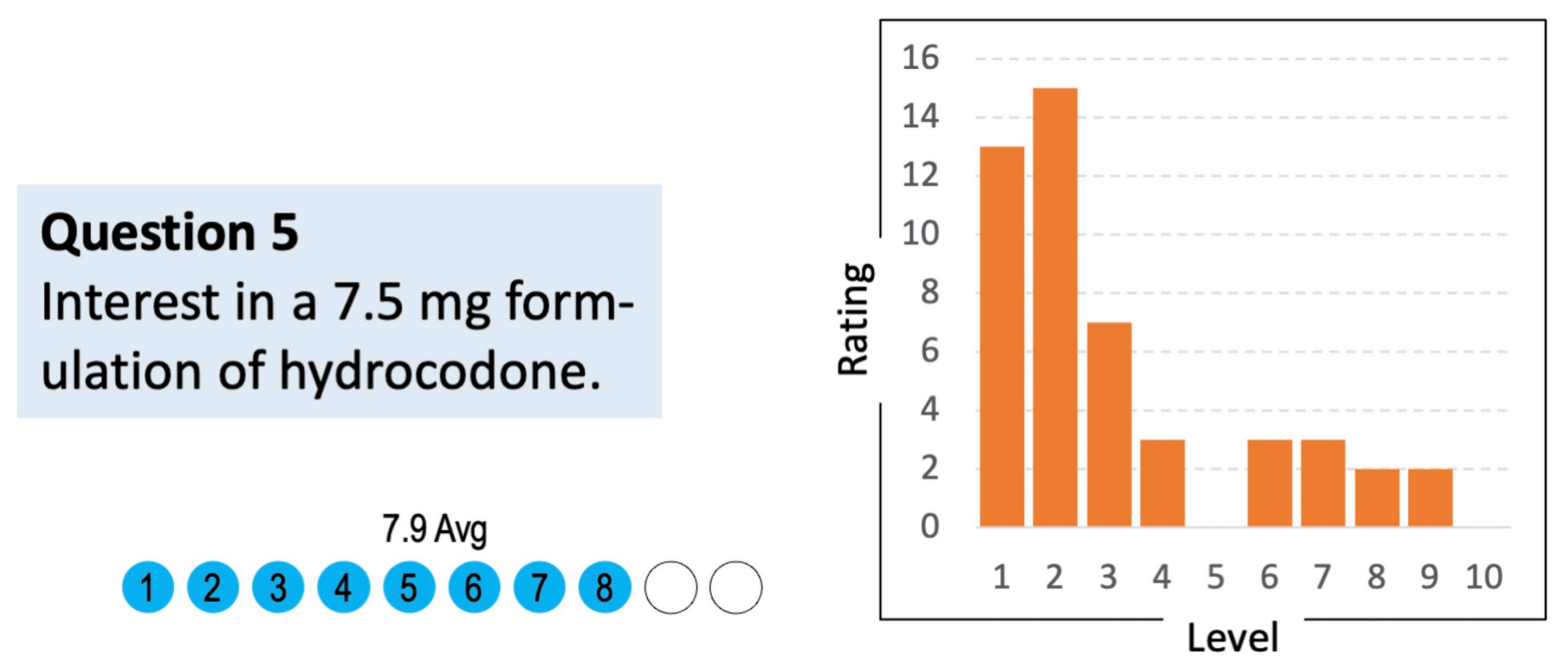
Average respondent interest in a 7.5 mg hydrocodone formulation (N = 48) Respondents indicated their level of interest on a scale of 1 (“no interest”) to 10 (“extremely interested”)

Respondents expressed high overall interest in the availability of a lower hydrocodone dose. The average rating across all providers was 7.9 out of 10, with many respondents selecting scores at the upper end of the scale (9 or 10) (Figure 5). These results suggest a strong HCP desire for a lower dose option that could provide greater flexibility in tailoring therapy to individual patient needs.

Question 6: Which option most closely represents your perceived benefit that a 7.5 mg hydrocodone dose could provide to patients if available?

**Figure 6:**
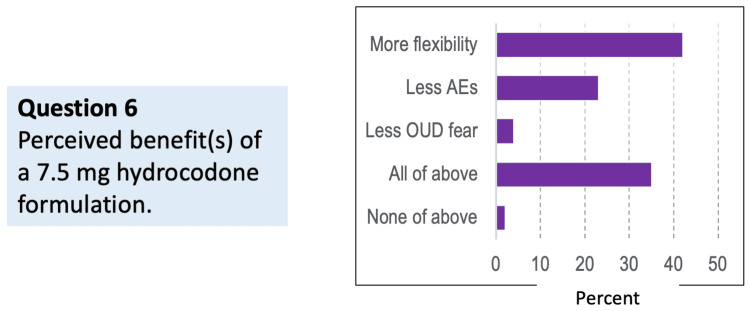
Perceived benefit(s) of a 7.5 mg hydrocodone formulation (N = 48) “Flexibility” refers to dosing options AEs: adverse effects, OUD: opioid use disorder

Respondents identified several potential benefits of introducing a lower hydrocodone formulation, including more flexible dosing, fewer adverse events, and less fear of causing opioid use disorder (OUD). Importantly, many respondents felt that all listed benefits would apply, reinforcing the perceived clinical appeal of a lower-dose hydrocodone option (Figure 6).

## Discussion

This convenience sample survey [14] of HCP attendees at the annual (2025) PAINWeek meeting offers insight into current analgesic prescribing practices involving opioids and HCP attitudes about a postulated lower dose option of an opioid formulation (hydrocodone as an example). With regard to prescribing practices, most respondents reported that they actively prescribe analgesics, with prescribing patterns reflecting a balance of non-opioid, opioid, and combination opioid + non-opioid therapies [15]. This suggests that providers are currently implementing multimodal approaches to pain management, consistent with the pharmacologic advantages (including additive or synergistic interactions) [16,17], and best practice recommendations [18,19].

Hydrocodone remains a widely prescribed opioid for analgesia, principally in the United States [20], both as a standalone therapy and in combination with non-opioids [21]. The reliance on combination prescribing highlights HCP efforts to optimize pain relief while attempting to minimize opioid-related risks, consistent with previous reports [22]. However, the current results, based on strong respondent response ratings, indicate that HCPs see meaningful value in having a lower-dose opioid (hydrocodone) option available. Providers emphasized the importance of dose flexibility and reduced adverse events, with many reporting that all listed benefits would apply. This signals a perceived clinical gap that a 7.5 mg hydrocodone formulation could fill.

The results of the survey indicated the existence of a high interest on the part of the HCPs (viz., physicians, nurse practitioners, and physician assistants) in a lower-dose hydrocodone formulation. The respondents identified that the key perceived benefits of the availability of a lower dose were more flexible dosing, fewer adverse events, and less fear of causing opioid use disorder. About a third indicated that all three were perceived as desirable benefits. These findings suggest that expanding opioid dosing options could help support individualized pain management strategies across a range of specialties. If further validated, these results might provide insight into the desirability on the part of HCPs for more flexible, specifically lower, dosing options for the prescription of opioid analgesics.

These findings also appear to identify an opportunity to improve individualized pain management by expanding opioid (hydrocodone) dosing options. The available dosing formulations are dictated by positive results, i.e., statistically greater effect than placebo in clinical trials [23]. The available clinical dosing regimens consist of the available formulations and combinations thereof. However, due to differences in the demographics of study participants and distribution of patient responsiveness, the lowest approved dose can be too high for individual patients [24]. The consistent interest expressed across specialties in the current survey underscores that the potential benefits of a lower-dose option for hydrocodone would be widely viewed as desirable in clinical practice.

This survey has several limitations that should be considered when interpreting the findings. The first limitation was the sample size. With 48 participants, the sample size was relatively small and may not reflect the full spectrum of clinical practice patterns across different regions or specialties. The second limitation was the use of convenience sampling; respondents were recruited in person at PAINWeek 2025, which may limit the generalizability of results to the broader population of HCPs. The third limitation was self-reported responses; the responses are based on HCP self-reporting and may be influenced by recall or social desirability bias. The final limitation included the limited scope. The survey focused on an individual opioid (hydrocodone) as an example in order to assess opioid analgesic prescribing and interest in a postulated new lower formulation; broader aspects of pain management practices were not explored. A reviewer pointed out that Question 6 is framed in a leading manner. By only offering positive clinical outcomes (“More flexibility,” “Less AEs,” “Less OUD fear,” and “All of above”) and a passive “None of above” option, the survey might have artificially driven respondents toward validating the product, since there were no options provided for potential drawbacks, such as “inadequate analgesia,” “increased pill burden,” or “no clinical difference.”

Despite these limitations, we believe the survey provides valuable preliminary insights into healthcare provider perspectives and highlights meaningful possible ways to enhance clinical flexibility in pain management.

## Conclusions

This report summarizes the design, implementation, and insights obtained from a convenience survey of attendees conducted at the 2025 PAINWeek annual meeting. The objective of the survey was to assess the perspectives of a pilot convenience sample of HCPs and their interest in the postulated lower-dose option of opioid analgesics, using the specific example of a potential lower-dose hydrocodone formulation (viz., 7.5 mg). The survey respondents represented a small but diverse sample of clinical specialties and professional backgrounds. It captured information on HCP demographics, current prescribing practices for analgesics, interest in a potential lower (hydrocodone) formulation, and their perceived benefits if this lower dosage strength was to be introduced. Based on the results, it appears that further research in larger and more diverse provider populations is warranted to confirm these insights and, if confirmed, guide potential recommendations.

In summary, this pilot convenience survey of HCPs involved in opioid prescribing demonstrates that there might be a strong clinical interest in the availability of a lower opioid dose formulation. While providers currently use a mix of non-opioid, opioid, and combination therapies, the postulated addition of a lower-dose single-entity opioid option was widely viewed by this group as beneficial for improving dosing flexibility, minimizing adverse events, and better aligning treatment with individual patient needs.
